# Validation of Digital Slide Scanning and a Convolutional Neural Network for the Detection of Intestinal Parasites in Human Stool Samples

**DOI:** 10.3390/diagnostics15232974

**Published:** 2025-11-24

**Authors:** Céline Büschlen, Daniel Rotzer, Nadine Sidler, Ha Thu Trang Nguyen, Alexander Oberli

**Affiliations:** Institute for Infectious Diseases, University of Bern, Friedbühlstrasse 25, 3001 Bern, Switzerland

**Keywords:** intestinal parasites, digital microscopy, artificial intelligence, convolutional neural network, deep learning, screening, protozoan parasites, helminth eggs

## Abstract

**Background**: Recent studies have shown that digital microscopy (DM) combined with a convolutional neural network (CNN) model is a valid approach for detecting intestinal protozoa and helminth ova or larvae in both trichrome-stained and wet-mount stool preparations. This study evaluated the diagnostic performance of a DM/CNN workflow for routine detection of intestinal parasites in a clinical microbiology laboratory. **Methods**: A clinical validation was conducted using the Grundium Ocus 40 scanner combined with the Techcyte Human Fecal Wet Mount (HFW) algorithm. The system was evaluated on (a) 135 reference samples and (b) 208 routine clinical samples submitted for intestinal parasite testing. Analytical sensitivity, precision, and limit of detection (LOD) were assessed. **Results**: For reference samples, the DM/CNN workflow achieved a positive slide-level agreement of 97.6% (95% CI: 94.4–100%), following a confidence threshold adjustment for *Schistosoma mansoni*, and a negative agreement of 96.0% (95% CI: 86.6–98.9%) compared with light microscopy (LM). Dilution series with reference samples revealed slightly lower analytical sensitivity of the DM/CNN at higher dilutions. Both intra- and inter-run precision studies demonstrated high reproducibility and stability. In prospective testing on 208 routine samples, overall agreement between DM/CNN and LM was 98.1% (95% CI: 95.2–99.2%) with a Cohen’s Kappa coefficient of *κ* = 0.915. Minor discrepancies involved *Blastocystis* spp., with DM/CNN showing slightly higher sensitivity. **Conclusions**: For the first time, we show that the combination of the Grundium Ocus 40 scanner and the Techcyte HFW algorithm provides a reliable, low-throughput screening solution that can effectively assist diagnostic technicians by pre-classifying putative parasitic structures for targeted expert review. Despite its lower throughput, the system substantially reduces the manual review process and simplifies the parasitological workflow. Implementation in a clinical microbiology laboratory requires extensive site-specific validation to account for differences in sample processing and imaging conditions. Moreover, optimization of confidence thresholds for specific classifiers is essential to ensure consistent analytical performance across different laboratory settings.

## 1. Introduction

Intestinal parasitic infections affect an estimated 3.5 billion people globally, with the highest prevalence in tropical and subtropical regions where climatic and sanitary conditions favor transmission [1]. A variety of diagnostic methods, such as sedimentation, flotation, or formalin-ethyl acetate, followed by light microscopy, are routinely employed to detect intestinal parasites [2]. Each method, however, differs in sensitivity, turnaround time, and diagnostic spectrum, depending largely on laboratory infrastructure and expertise.

Despite advantages in antigen-, antibody-, and molecular-based diagnostics, manual microscopic examination of concentrated stool samples remains the gold standard for identifying intestinal protozoa and helminths [3]. Nevertheless, this approach is labor-intense, time-consuming, highly reliant on the expertise and training of the microscopist, and subject to operator variability. This underscores the need for standardized, traceable, and cost-effective diagnostic alternatives that reduce workload while maintaining high accuracy.

Recent developments in machine learning (ML), a subset of artificial intelligence (AI), have gained traction in clinical microbiology, demonstrating the potential to enhance diagnostic accuracy, reduce turnaround time, and improve cost efficiency [4,5,6]. ML-based diagnostic tools are now successfully applied for assistance in antimicrobial susceptibility testing [7], Gram-stain interpretation [8], detection of methicillin-resistant *Staphylococcus aureus* (MRSA) [9] and vancomycin-resistant *Enterococcus* (VRE) in screening cultures [10], as well as *Mycobacterium tuberculosis* in sputum smears [11,12].

In parasitology, ML has been explored for the automated detection of *Plasmodium* spp. in Giemsa-stained blood smears in clinical microbiology laboratory settings [13] and in malaria-endemic field settings [14], *Schistosoma haematobium* eggs in urine sediments [15], *Schistosoma mansoni* eggs in stool samples [16], and the most common soil-transmitted helminths (including *Ascaris lumbricoides*, *Trichuris trichiura* and hookworms) in stool samples [17]. Mathison et al. first validated a digital microscopy (DM) and convolutional neural network (CNN) model for detecting intestinal protozoa in trichrome-stained specimens [18]. The study investigated the feasibility of employing a CNN model to automatically screen trichrome-stained slides and to flag potential parasitic structures for subsequent manual verification. In comparison to light microscopy (LM) performed by human technologists, the integrated workflow, combining digital slide scanning and CNN-based analysis demonstrated superior analytical sensitivity while maintaining comparable slide-level diagnostic accuracy in determining presence or absence of target parasites. A subsequent study extended this approach using a similar CNN model for the detection of intestinal parasites in fixed fecal wet mounts, achieving >90% slide-level agreement with LM and identifying additional true positive cases after discrepant analysis [19].

Building upon these findings, the present study aimed to clinically validate the Grundium Ocus 40 scanner in combination with the Techcyte Human Fecal Wet Mount (HFW) algorithm for detecting intestinal parasites in human wet-mount stool preparations, representing the first prospective study to validate this specific scanner–algorithm combination in a routine diagnostic setting. The study compared DM/CNN-based pre-classification/classification with LM as the reference standard and evaluated analytical performance, including accuracy, precision, and reproducibility, using both reference and prospective clinical samples. To our knowledge, this is the first study to demonstrate the application of such an approach within a daily routine diagnostic setting.

## 2. Material and Methods

### 2.1. Ethics Statement

The cantonal ethical committee of Bern, Bern, Switzerland, exempted the validation study after clarification of responsibility (Req-2024-00087) because the project was not subject to the Human Research Act. All healthcare-related data were anonymized.

### 2.2. Study Design and Sample Collection

The validation study consisted of two complementary parts. First, to assess the diagnostic accuracy of the AI-assisted algorithm relative to expert-based light microscopy (LM), a reference panel of 85 human stool sediment samples with confirmed parasitic infections was established. The panel was designed to include all relevant target parasite species detectable by the algorithm, with at least three positive specimens per organism/class whenever available. Due to their rarity in clinical practice, sufficient specimens could not be included for *Clonorchis sinensis*/*Opisthorchis* spp. (*n* = 0), *Paragonimus* spp. (*n* = 0), *Paracapillaria philippinensis* (*n* = 1), *Schistosoma japonicum/Schistosoma mekongi* (*n* = 0), *Dientamoeba fragilis* (*n* = 0), *Entamoeba hartmanni* (*n* = 1) and *Entamoeba polecki* (*n* = 0).

Additionally, 50 confirmed parasite-negative stool samples were included as controls.

**Figure 1:**
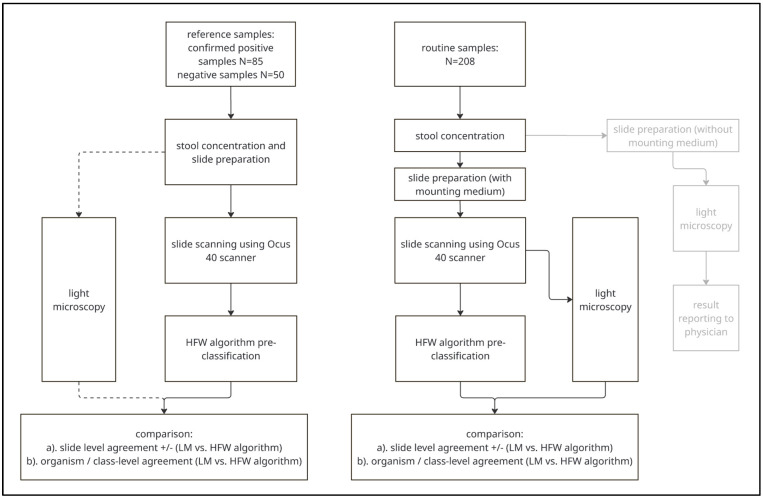
Flowchart detailing the methodical sequence for both reference samples and routine diagnostic samples. The light grey content indicate a different workflow and is not covered in the manuscript.

In the second part, to evaluate performance under routine diagnostic conditions, all stool samples submitted to the Institute for Infectious Diseases, University of Bern, Bern, Switzerland, for intestinal parasite testing over a three-month period (*n* = 208) were analyzed in parallel using both LM and the DM/CNN approach. The overall study design and analytical workflow for both reference samples and routine diagnostic samples are illustrated in Figure 1.

### 2.3. Sample Preparation

All stool samples were received in sodium-acetate-acetic acid-formalin (SAF) fixative tubes to preserve morphological integrity during transport and processing. Potentially present parasitic structures were concentrated using the StorAX SAF filtration device (AxonLab, Baden, Switzerland) following the manufacturer’s instructions. In brief, the procedure included stool homogenization in SAF, filtration, addition of Triton^TM^X-100 and ethyl acetate, centrifugation at 505× *g* for 10 min, and removal of the supernatant to obtain sediment for microscopy.

For comparison analysis, the Mini Parasep SF device (Apacor, Workingham, UK) was evaluated as an alternative concentration method. The final volume was 10 mL for the StorAX SAF device and 3–5 mL for the Mini Parasep SF device.

### 2.4. Slide Preparation

Slides were prepared as described by Mathison et al., 2025 [19]. Briefly, 15 µL of stool sediment was mixed with 15 µL of mounting medium composed of Lugol’s iodine and glycerol in phosphate-buffered saline (PBS) on a 75 × 25 mm glass slide and covered with a 22 × 22 mm glass coverslip. The mounting medium volume was increased up to 20 µL, depending on sample viscosity. To prevent drying, all slides were sequentially prepared, scanned and examined by LM.

### 2.5. Light Microscopy

Manual light microscopy served as the diagnostic gold standard. All reference samples were examined by technologists with over 20 years of parasitology experience prior to further analysis, while routine diagnostic samples were read by technologists with at least 5 years of experience. All examinations were performed fully blinded to the DM/CNN results, and during the initial analysis of the prospective cohort, technologists were also blinded to each sample’s reference status. Slides were examined using an Olympus BX45 microscope (Olympus, Tokyo, Japan) at 100× magnification for detection of helminth ova and larvae, and at 400× magnification for identification of protozoan cysts and trophozoites.

### 2.6. Scanning/Digital Microscopy

Slides were scanned using the Grundium Ocus 40 slide scanner (Grundium Oy, Tampere, Finland) equipped with a 20× 0.75 NA objective. The 22 × 22 mm coverslip area was captured at an effective 40× magnification (0.25 microns per pixel) across two focal planes. Scans were saved as individual FOVs in JPEG format onto internal scanner storage for upload to Techcyte (Techcyte Inc., Orem, UT, USA). After each scan, the focal plane was visually verified to ensure image quality. Reference slides were loaded in random order, without grouping by target parasite, to minimize potential bias in image analysis.

### 2.7. Software/Classification Algorithm

Detection and classification of target parasites were performed using the Techcyte Human Fecal Wet Mount (HFW) algorithm, version 1.0 (Techcyte Inc., Orem, UT, USA). The algorithm analyzed digital slide images to determine the presence or absence of target parasites (pre-classification) and proposed organism/class-level identifications by labeling image regions accordingly. The model was trained to recognize a broad range of helminths and protozoa, including *Ascaris lumbricoides*, *Clonorchis sinensis*/*Opisthorchis* spp., *Dibothriocephalus latus*, *Enterobius vermicularis*, *Fasciola* spp./*Fasciolopsis buski*, hookworm/*Trichostrongylus*, *Hymenolepsis diminuta*, *Paragonimus* spp., *Paracapillaria philippinensis*, *Rodentolepsis nana*, *Schistosoma mansoni*, *Schistosoma japonicum*/*Schistosoma mekongi*, *Strongyloides* spp., *Taenia* spp., *Trichuris trichiura*, *Balantoides coli*, *Blastocystis* spp., *Chilomastix mesnili* cysts, *Cyclospora* spp., *Cystoisospora belli*, *Endolimax nana* cysts, *Entamoeba* species (*E. coli*, *E. histolytica*/*dispar*, *E. polecki*), *Giardia duodenalis*, *Iodamoeba buetschlii* cysts, Miscellaneous Small Protozoans (trophozoites of *Chilomastix mesnili*, *Dientamoeba fragilis*, *Endolimax nana*, *Iodamoeba buetschlii*, and cysts and trophozoites of *Entamoeba hartmanii*). Each organism prediction received an uncalibrated confidence score ranging from 0 to 1. Predictions above an optimized threshold were displayed for manual review. Thresholds were adjusted to minimize false negatives while maintaining acceptable false-positive results, thereby maximizing slide-level sensitivity and workflow efficiency. The software presented pre-classification results (positive, negative) and ranked putative organisms/classes in descending order of confidence. All labeled regions were manually reviewed by a technologist and specimens were classified as positive if at least one target parasite was confirmed. All positive specimens underwent additional verification by LM by a technologist with over 20 years of parasitology experience.

### 2.8. Software Adjustments

Performance optimization revealed that lowering the confidence threshold for *Schistosoma mansoni* from 0.92 to 0.7 significantly improved detection performance by increasing the number of slides with correct pre-classification from one to four out of five (Table 1). The threshold adjustment was informed not only by the five *S. mansoni* reference samples but also by evaluations across additional slides examined during parameter calibration. This adjustment was incorporated into an updated algorithm (Techcyte HFW version 1.0.1), ensuring that all subsequent users benefit from this refinement without additional configuration. This finding underscores the importance of monitoring and addressing AI model drift when algorithms are applied to data from new laboratory settings.

### 2.9. Assay Accuracy

Assay accuracy was assessed across 135 reference samples and 208 clinical samples using two performance metrics: (a) slide-level agreement (parasite detected vs. not detected) and (b) organism/class-level agreement. Discrepant results at either level were reevaluated by preparing and reviewing a new slide of the initial specimen by LM. Slide-level agreements were defined as follows:

True positive (TP): target parasite correctly detected by the algorithm.

True negative (TN): no target parasite present and none detected (or insufficient flagged to trigger manual review by LM).

False positive (FP): target parasite flagged by the algorithm but not confirmed by LM.

False negative (FN): target parasite present but not detected by the algorithm.

### 2.10. Precision

Precision was evaluated using reference stool samples containing *Ascaris lumbricoides*, *Giardia duodenalis*, and a parasite-negative control. Intra-run precision was assessed by scanning and analyzing each specimen three times within the same day. Inter-run precision was determined by preparing, scanning and analyzing new slides of each specimen on four additional days, for a total of five testing days. Detection of the target parasite at the slide level was recorded for each replicate.

### 2.11. Limit of Detection

The analytical sensitivity of both diagnostic methods was determined using serial dilutions of two reference samples containing *A. lumbricoides* eggs and *Blastocystis* spp. cysts. Samples with high initial parasite loads were chosen to minimize discrepancies at higher dilutions. Stool sediments were diluted in SAF fixative up to 1:512, and five slides were prepared per dilution (including undiluted samples) using 20 µL of mounting medium and 15 µL of sample. All slides were randomized, independently examined by two experienced technologists (>5 years parasitology experience), and subsequently scanned to prevent drying. Dilution series were distributed among routine diagnostic samples to minimize observer bias. A result was considered positive when at least one target parasite was clearly identified, and the limit of detection (LOD) was defined as the highest dilution at which ≥4 of 5 replicates tested positive, corresponding to an 80% detection probability consistent with the CLSI EP12 probability-of-detection framework for qualitative assays.

### 2.12. Statistical Analyses

Statistical analyses were performed using R software (version 4.3.3; R Foundation for Statistical Computing, Vienna, Austria).

## 3. Results

### 3.1. Comparison of Filtration Units

A comparative evaluation of two filtration devices was performed, as Techcyte recommends the Mini Parasep SF device, whereas the Institute for Infectious Diseases (University of Bern, Bern, Switzerland) routinely uses the StorAX SAF filtration device. In total, 20 reference stool samples containing various target parasites and 10 target parasite-negative samples were analyzed using both devices (Table S1). All target parasites were detected by both LM and the DM/CNN workflow, and all negative samples tested negative across both methods. Using LM, comparable parasite counts were observed in 8 of the reference samples, while the StorAX SAF device yielded higher parasite counts in 12 samples, indicating slightly superior concentration efficiency.

### 3.2. Accuracy

Slide-level: Slide-level agreement (pre-classification as positive or negative for target parasite) was used as the primary accuracy metric and calculated for each target parasite (Table 1). The predefined acceptance criterion of ≥95% agreement was met for 21 target parasites, with the exception of *Schistosoma mansoni* (1/5, 20%) and *Strongyloides* spp. (2/3, 66.7%). Overall, the DM/CNN workflow achieved a positive slide-level agreement of 94.1% (95% CI: 89.1–99.1%) and a negative slide level of 96.0% (95% CI: 86.6–98.9%), with two false positive pre-classifications for *Blastocystis* spp.

After lowering the confidence threshold for *S. mansoni* from 0.92 to 0.7, scans were re-analyzed using the updated HFW algorithm (version 1.0.1). This classifier adjustment increased correct pre-classification for 4/5 *S. mansoni* reference specimens, resulting in an overall positive slide-level agreement of 97.6% (95% CI: 94.4–100%), while the negative agreement remained unchanged.

Organism/class level: At the organism/class level, manual verification of all pre-classified positive samples yielded an overall agreement of 83.5%, which improved to 87.0% with algorithm version 1.0.1. Correct classification was achieved for 17 target parasites, whereas misclassification occurred primarily for *S. mansoni* (0/5, 0%; 3/5, 60% with v1.0.1), *Strongyloides* spp. (0/3, 0%), *Chilomastix mesnili* (2/3, 66.7%), *Endolimax nana* (3/5, 60%), *Entamoeba histolytica/dispar* (3/5, 60%), *Giardia duodenalis* (4/5, 80%).

### 3.3. Precision

Precision was evaluated using reference samples containing *Ascaris lumbricoides*, *Giardia duodenalis*, and a parasite-negative stool specimen. For intra-run precision, each slide was scanned and analyzed three times on the same day, with consistent detection of both target parasites in all replicates (Table S2). For inter-run precision, new slides of each sample were prepared and analyzed on five consecutive days (Table S3). The HFW algorithm consistently detected the target parasites across all testing days, demonstrating high analytical repeatability and reproducibility.

### 3.4. Limit of Detection

The analytical sensitivity of the LM and DM/CNN workflow was compared using serial dilutions (up to 1:512) of stool samples containing *Ascaris lumbricoides* ova and *Blastocystis* spp. cysts (Table 2). The limit of detection (LOD) was defined as the highest dilution step at which ≥4 of 5 slides remained positive. For *A. lumbricoides*, the LOD was 1:128 for LM and 1:64 for DM/CNN. For *Blastocystis* spp., LM detected cysts in all slides up to 1:128 and the LOD was 1:256, whereas the DM/CNN workflow failed to detect cysts at 1:256, yielding a LOD of 1:64. To determine whether the observed differences in detection rates were statistically significant, Fisher’s exact tests were applied at the dilution steps where the two methods diverged. For *A. lumbricoides*, none of the differences at 1:64, 1:128, or 1:256 reached statistical significance (all *p* = 0.5), indicating comparable detection performance across methods. For *Blastocystis* spp., a significant difference was observed only at the 1:256 dilution (*p* = 0.024), whereas divergence at 1:128 and 1:512 was not significant (both *p* = 0.083). Overall, despite slightly lower LOD values for DM/CNN, statistically significant differences were limited to one dilution step for *Blastocystis* spp., and none for *A. lumbricoides.*

### 3.5. Prospective Testing

To assess the applicability of the DM/CNN workflow under routine diagnostic conditions, 208 stool samples from patients with suspected intestinal parasitic infections were analyzed in parallel by LM and the DM/CNN system over a three-month period. Results from both workflows matched in 204 samples (179 negative, 25 positive), corresponding to a pre-classification slide-level agreement of 98.1% (95% CI: 95.2–99.2%) and a Cohen’s kappa coefficient of *κ* = 0.915, indicating excellent agreement (Table 3).

Discrepancies were observed in three samples (sample ID 163, 195, and 172), in which *Blastocystis* spp. were not initially detected by LM but were pre-classified as positive by DM/CNN (Table 4). Manual review and re-examination of new slides by LM confirmed the presence of *Blastocystis* spp. in all three samples, although parasite numbers were low and degeneration of the structure was observed. In contrast, DM/CNN failed to detect *Blastocystis* spp. in sample 177, which was later confirmed positive by LM, including a slide without mounting medium (routine diagnostic workflow). These findings suggest slightly higher sensitivity of the DM/CNN workflow for *Blastocystis* spp. detection in low-density or morphologically degraded specimens.

## 4. Discussion

Despite the recent development and commercial availability of sensitive molecular and antigen-based diagnostic assays for intestinal protozoa and helminths, morphological examination of stool samples by microscopy remains the diagnostic gold standard. Light microscopy (LM) offers a broad diagnostic scope, enabling the detection of a wide range of protozoa and helminths. However, this approach is resource-intensive, time-consuming, variable in sensitivity, and highly dependent on trained personnel. In low-prevalence settings, intestinal protozoan and helminths account for a small proportion of infectious diseases, yet the diagnostic workload remains disproportionately high. Although workflow optimization can improve sample processing efficiency, large numbers of technologists are still required to manually screen slides for low-abundance parasites—a subjective and time-consuming task that could be efficiently performed using machine learning (ML)-based image analysis (including sensitivity optimization over class/organism specificity).

Given the increasing challenges in maintaining microscopy and morphology competence in parasitology diagnostics [20], the integration of ML-assisted screening could allow technologists to bypass routine negative screening and focus on samples pre-classified as positive, thereby enhancing their morphological diagnostic proficiency. Recent studies have demonstrated the feasibility of combining digital slide scanning with convolutional neural network (CNN)-based algorithms for both trichrome-stained [18] and wet-mount stool samples [19], marking a promising step towards automated triaging of presumptive positive slides for confirmatory manual review.

In this study, we first validated a CNN-based digital microscopy workflow for the detection of intestinal parasites in sodium acetate-acetic acid-formalin (SAF)-preserved stool samples and evaluated its practical use in a routine diagnostic microbiology laboratory with low to moderate throughput. In our implementation, throughput was mainly limited by the Grundium Ocus 40 scanner, which can process only one slide at a time, making the workflow more suitable for low- to moderate-volume settings. To preserve morphological integrity already during transport, stool samples were collected in SAF fixative tubes and processed using either the StorAX SAF or Mini Parasep SF filtration devices. Both devices supported efficient parasite detection, but the StorAX SAF generally yielded higher parasite loads in 12 of 20 reference samples, likely due to its larger input volume. Owing to the superior analytical yield observed, the StorAX SAF device was subsequently used for all further analyses conducted in the course of the study.

The primary performance metric for the clinical validation was slide-level accuracy. In the implemented workflow, slides containing putative target parasites were flagged by the HFW algorithm for manual confirmation by a trained technologist using digital scans, while unflagged slides could be rapidly reviewed and confirmed as negative, thereby streamlining the diagnostic workflow. Although the algorithm occasionally flagged non-parasitic structures that morphologically resembled protozoa or helminths, these false positives were easily resolved during manual review, commonly involving *Blastocystis* spp. The initial positive slide-level agreement between digital microscopy (DM) with CNN (DM/CNN) and LM was 94.1% (95% CI: 89.1–99.1%), below the predefined ≥95% concordance criterion. This shortfall was primarily due to lower performance for *Schistosoma mansoni* and *Strongyloides* spp. Adjustment of the *S. mansoni* classifier cutoff and implementation of HFW algorithm version 1.0.1 improved pre-classification agreement to 80% for *S. mansoni* and 97.6% (CI: 94.4–100%) overall, thus fulfilling the concordance requirement. Lowering the *S. mansoni* confidence threshold to 0.7 further enhanced sensitivity without affecting negative agreement, underscoring the need for laboratory-specific optimization of classifier thresholds. Detection of *Schistosoma* spp. remains challenging, as ova are frequently obscured by debris or artifacts. Combining DM/CNN and LM for patients with clinical or geographical suspicion of *Schistosoma* infection may therefore enhance diagnostic yield. Similarly, *Strongyloides stercoralis* failed to meet the ≥95% agreement threshold, mainly due to scanner limitations in focal plane detection, which impaired visualization of larval morphology. Because accurate recognition of *Strongyloides* requires precise imaging, future improvements in scanner optics, focal plane control, and adoption of volumetric scanning are expected to enhance detection performance.

At the organism/class level, the HFW algorithm achieved encouraging predictive performance (83.5% and 87.0% for version 1.0.1), though several parasites such as *S. mansoni*, *Strongyloides* spp., *Chilomastix mesnili*, and *Entamoeba histolytica/dispar* showed lower accuracies. These discrepancies highlight the difficulty of distinguishing morphologically similar or rare parasites using automated image analysis and emphasize the need for algorithm refinement using larger, well-characterized training datasets. Nonetheless, the rarity of some target parasites limits the availability of reference material. Importantly, the algorithm is intended to function as a high-sensitivity screening system for gastrointestinal parasites, deliberately optimizing sensitivity at the expense of organism-level specificity. As a result, all algorithm-flagged positive samples require downstream verification—either through expert morphologic assessment of the digital images or confirmatory LM of the sample—to ensure accurate organism/class level identification.

Precision testing confirmed high intra- and inter-run reproducibility, supporting the workflow’s reliability. In the limit of detection (LOD) analysis, LM and the DM/CNN workflow showed comparable analytical performance throughout most dilution steps, with statistically significant differences observed only at a single dilution step for *Blastocystis* spp. and none for *A. lumbricoides*. At extreme dilutions, image quality was strongly affected by reduced morphological reference structure, limiting the scanner’s ability for focal plane determination. Additionally, potential selection bias may have favored LM performance, as technologists may have recognized diluted samples and examined them more thoroughly. While DM/CNN offers substantial advantages in automation and throughput, its overall sensitivity is only marginally lower than LM and largely comparable across the evaluated dilution range. These findings differ from previous reports using trichrome- and wet mount-based CNN workflows, which achieved lower LODs relative to LM [18,19]. Such differences likely arise from variations in sample type, scanner type, parasite abundance, algorithm version, as well as differences in the study design and human verification procedures.

Prospective clinical testing of routine stool samples further demonstrated the robustness and practical value of the DM/CNN workflow. A pre-classification slide-level agreement of 98.1% (95% CI: 95.2–99.2%) and Cohen’s kappa coefficient *κ* = 0.915 indicated excellent concordance with LM. Importantly,

DM/CNN identified low-abundance *Blastocystis* spp. cases missed by routine LM, though one low-density sample was not detected by DM/CNN. Manual review of discrepant cases revealed parasite degeneration and slide preparation artifacts as contributing factors, emphasizing that both DM/CNN and LM may face challenges in detecting degraded or low-abundance parasites.

This study had certain limitations related to sample diversity and representation. Several rare parasites, including *Clonorchis sinensis*/*Opisthorchis* spp., *Paragonimus* spp., *Schistosoma japonicum/Schistosoma mekongi*, *Dientamoeba fragilis*, and *Entamoeba polecki*, were either absent or underrepresented in the dataset, thereby limiting the generalizability of the findings for these target parasites. Moreover, this limited representation significantly constrains the ability to assess robust performance across the algorithm’s entire diagnostic spectrum. Additionally, the number of positive reference samples per class/organism was small (≥3 by study design), representing a key statistical limitation that reduces the precision and robustness of the corresponding performance. In addition, the diagnostic performance of the DM/CNN workflow was influenced by technical characteristics of the Grundium Ocus 40 scanner, particularly focal plane variability, which can compromise digital image quality and hinder accurate parasite detection by the HFW algorithm.

In summary, this study provides strong evidence for the integration of a digital microscopy and convolutional neural network (DM/CNN)-based workflow, supplemented by manual review, into routine clinical microbiology diagnostics. This approach enables reliable exclusion of negative samples and enhances overall diagnostic efficiency. The DM/CNN approach achieved excellent slide-level agreement with LM, high precision, and improved sensitivity in routine diagnostic samples. While some limitations persist—particularly in focal plane determination and sensitivity for certain target parasites—ongoing advances in scanning technology and algorithm retraining with larger datasets are expected to further enhance performance. To our knowledge, this is the first study to document the successful implementation of such a semi-automated image analysis workflow for wet-mount stool specimens within daily routine diagnostic practices. Together, these findings underscore the potential of AI-assisted microscopy to advance diagnostic standardization, scalability, and automation in clinical microbiology, demonstrating that the current DM/CNN workflow serves as an effective screening and triage tool that enables technologists to focus on AI-flagged samples and achieve higher overall diagnostic efficiency.

## Figures and Tables

**Table 1:** Target parasites and accuracy results for reference stool samples. Results obtained using HFW algorithm version 1.0.1 are displayed in orange color. Species with different background color belong to different groups.

Category (Species)	Number of Reference Sample/s	Slide-Level Agreement	Organism/Class-Level Agreement
*Ascaris lumbricoides*	5	5/5 (100%)	5/5 (100%)
*Clonorchis sinensis/Opisthorchis* spp.	0	-	-
*Enterobius vermicularis*	3	3/3 (100%)	3/3 (100%)
*Fasciola* spp./*Fasciolopsis buski*	3	3/3 (100%)	3/3 (100%)
*Diphyllobothrium latum*	5	5/5 (100%)	5/5 (100%)
Hookworm/*Trichostrongylus*	5	5/5 (100%)	5/5 (100%)
*Hymenolepsis diminuta*	3	3/3 (100%)	3/3 (100%)
*Paragonimus* spp.	0	-	-
*Paracapillaria philippinensis*	1	1/1 (100%)	1/1 (100%)
*Rodentolepsis nana*	3	3/3 (100%)	3/3 (100%)
*Schistosoma mansoni* *Schistosoma mansoni (HFW algorithm v. 1.0.1)*	5	1/5 (20%) 4/5 (80%)	0/5 (0%) 3/5 (60%)
*Schistosoma japonicum /Schistosoma mekongi*	0	-	-
*Strongyloides* spp.	3	2/3 (66.7%)	0/3 (0%)
*Taenia* spp.	3	3/3 (100%)	3/3 (100%)
*Trichuris trichiura*	3	3/3 (100%)	3/3 (100%)
*Balantioides coli*	3	3/3 (100%)	3/3 (100%)
*Blastocystis* spp.	5	5/5 (100%)	5/5 (100%)
*Chilomastix mesnili*	3	3/3 (100%)	2/3 (66.7%)
*Cyclospora* spp.	3	3/3 (100%)	3/3 (100%)
*Cystoisospora belli*	3	3/3 (100%)	3/3 (100%)
*Dientamoeba fragilis* (categorized as MSP)	0	-	-
*Endolimax nana*	5	5/5 (100%)	3/5 (60%)
*Entamoeba coli* (categorized as *Entamoeba* spp.)	5	5/5 (100%)	5/5 (100%)
*Entamoeba hartmanni* (categorized as MSP)	1	1/1 (100%)	1/1 (100%)
*Entamoeba histolytica/dispar* (categorized as *Entamoeba* spp.)	5	5/5 (100%)	3/5 (60%)
*Entamoeba polecki* (categorized as *Entamoeba* spp.)	0	-	-
*Giardia duodenalis*	5	5/5 (100%)	4/5 (80%)
*Iodamoeba buetschlii*	5	5/5 (100%)	5/5 (100%)
Overall agreement (HFW algorithm version 1.0) Overall agreement *(HFW algorithm v. 1.0.1)*	-	80/85 (94.1%) 83/85 (97.6%)	71/85 (83.5%) 74/85 (87.0%)

**Table 2:** Limit of detection comparison between the light microscopy (LM) and the digital microscopy/convolutional neural network (DM/CNN) approach.

	*Ascaris lumbricoides *		*Blastocystis* spp.	
Dilution	LM	DM/CNN	LM	DM/CNN
1:1	5/5	5/5	5/5	5/5
1:2	5/5	5/5	5/5	5/5
1:4	5/5	5/5	5/5	5/5
1:8	5/5	5/5	5/5	5/5
1:16	5/5	5/5	5/5	5/5
1:32	5/5	5/5	5/5	5/5
1:64	5/5	4/5	5/5	4/5
1:128	4/5	3/5	5/5	2/5
1:256	2/5	1/5	4/5	0/5
1:512	0/5	0/5	3/5	0/5

**Table 3:** Contingency table for comparison of the light microscopy (LM) and the digital microscopy/convolutional neural network (DM/CNN) approach using 208 routine diagnostic stool samples.

		DM/CNN	
		Positive	Negative
LM	positive	25	1
	negative	3	179

**Table 4:** Classification of positive routine diagnostic stool samples. P, positive; N, negative, MSP, miscellaneous small protozoans. Red color indicates false negative result or misclassification.

Sample ID	LM	DM/CNN	LM with Separate Slide w.o. Mounting Medium
14	P/*Blastocystis* spp.	P/*Blastocystis* spp.	P/*Blastocystis* spp.
34	P/*Blastocystis* spp.	P/*Blastocystis* spp.	P/*Blastocystis* spp.
35	P/*Blastocystis* spp.	P/*Blastocystis* spp.	P/*Blastocystis* spp.
36	P/*Blastocystis* spp.	P/*Blastocystis* spp.	P/*Blastocystis* spp.
40	P/*Blastocystis* spp.	P/*Blastocystis* spp.	P/*Blastocystis* spp.
51	P/*Blastocystis* spp.	P/*Blastocystis* spp.	P/*Blastocystis* spp.
56	P/*Blastocystis* spp.	P/*Blastocystis* spp.	P/*Blastocystis* spp.
62	P/*Blastocystis* spp.	P/*Blastocystis* spp.	P/*Blastocystis* spp.
77	P/*G. intestinalis*	P/*G. intestinalis*	P/*G. intestinalis*
85	P/*Blastocystis* spp.	P/*Blastocystis* spp.	P/*Blastocystis* spp.
109	P/*Blastocystis* spp.	P/*Blastocystis* spp.	P/*Blastocystis* spp.
113	P/*G. intestinalis*	P/*G. intestinalis*	P/*G. intestinalis*
117	P/*Blastocystis* spp.	P/*Blastocystis* spp.	P/*Blastocystis* spp.
118	P/*Blastocystis* spp.	P/*Blastocystis* spp.	P/*Blastocystis* spp.
127	P/*Blastocystis* spp.	P/*Blastocystis* spp.	P/*Blastocystis* spp.
151	P/*Blastocystis* spp.	P/*Blastocystis* spp.	P/*Blastocystis* spp.
159	P/*Blastocystis* spp., *C. mesnili*	P/*Blastocystis* spp., MSP	P/*Blastocystis* spp.
160	P/*Blastocystis* spp., *C. mesnili*	P/*Blastocystis* spp., MSP	P/*C. mesnili*
163	N	P/*Blastocystis* spp.	P/*Blastocystis* spp.
165	N	P/*Blastocystis* spp.	N
167	P/*Blastocystis* spp.	P/MSP	P/*Blastocystis* spp.
168	P/*Blastocystis* spp.	P/*Blastocystis* spp.	P/*Blastocystis* spp.
169	P/*Blastocystis* spp.	P/*Blastocystis* spp.	P/*Blastocystis* spp.
170	P/*G. intestinalis*	P/*G. intestinalis*	P/*G. intestinalis*
172	N	P/*Blastocystis* spp.	N
177	P/*Blastocystis* spp.	N	P/*Blastocystis* spp.
183	P/*Blastocystis* spp.	P/*Blastocystis* spp.	P/*Blastocystis* spp.
201	P/*Blastocystis* spp.	P/*Blastocystis* spp.	P/*Blastocystis* spp.
205	P/*E. coli*	P/*E. coli*	P/*E. coli*

## Data Availability

The original contributions presented in this study are included in the article/Supplementary Material. Further inquiries can be directed to the corresponding author.

## Supplementary Materials

The following supporting information can be downloaded at: https://www.mdpi.com/article/10.3390/diagnostics15232974/s1.

## Abbreviations

AI	artificial intelligence
LM	light microscopy
CNN	convolutional neural network
DM	digital microscopy
HFW	human fecal wet mount
LOD	limit of detection
ML	machine learning
SAF	sodium acetate formalin

## References

[1] Ahmed M. (2023). Intestinal Parasitic Infections in 2023. Gastroenterol. Res..

[2] Koontz F., Weinstock J.V. (1996). The approach to stool examination for parasites. Gastroenterol. Clin. N. Am..

[3] Garcia L.S., Arrowood M., Kokoskin E., Paltridge G.P., Pillai D.R., Procop G.W., Ryan N., Shimizu R.Y., Visvesvara G. (2018). Practical Guidance for Clinical Microbiology Laboratories: Laboratory Diagnosis of Parasites from the Gastrointestinal Tract. Clin. Microbiol. Rev..

[4] Topol E.J. (2019). High-performance medicine: The convergence of human and artificial intelligence. Nat. Med..

[5] Burns B.L., Rhoads D.D., Misra A. (2023). The Use of Machine Learning for Image Analysis Artificial Intelligence in Clinical Microbiology. J. Clin. Microbiol..

[6] Smith K.P., Kirby J.E. (2020). Image analysis and artificial intelligence in infectious disease diagnostics. Clin. Microbiol. Infect..

[7] Fader R.C., Weaver E., Fossett R., Toyras M., Vanderlaan J., Gibbs D., Wang A., Thierjung N. (2013). Multilaboratory study of the Biomic automated well-reading instrument versus MicroScan WalkAway for reading MicroScan antimicrobial susceptibility and identification panels. J. Clin. Microbiol..

[8] Smith K.P., Kang A.D., Kirby J.E. (2018). Automated Interpretation of Blood Culture Gram Stains by Use of a Deep Convolutional Neural Network. J. Clin. Microbiol..

[9] Faron M.L., Buchan B.W., Vismara C., Lacchini C., Bielli A., Gesu G., Liebregts T., van Bree A., Jansz A., Soucy G. (2016). Automated Scoring of Chromogenic Media for Detection of Methicillin-Resistant Staphylococcus aureus by Use of WASPLab Image Analysis Software. J. Clin. Microbiol..

[10] Faron M.L., Buchan B.W., Coon C., Liebregts T., van Bree A., Jansz A.R., Soucy G., Korver J., Ledeboer N.A. (2016). Automatic Digital Analysis of Chromogenic Media for Vancomycin-Resistant-Enterococcus Screens Using Copan WASPLab. J. Clin. Microbiol..

[11] Panicker R.O., Soman B., Saini G., Rajan J. (2016). A Review of Automatic Methods Based on Image Processing Techniques for Tuberculosis Detection from Microscopic Sputum Smear Images. J. Med. Syst..

[12] Zingue D., Weber P., Soltani F., Raoult D., Drancourt M. (2018). Automatic microscopic detection of mycobacteria in sputum: A proof-of-concept. Sci. Rep..

[13] Kuo P.-C., Cheng H.-Y., Chen P.-F., Liu Y.-L., Kang M., Kuo M.-C., Hsu S.-F., Lu H.-J., Hong S., Su C.-H. (2020). Assessment of Expert-Level Automated Detection of Plasmodium falciparum in Digitized Thin Blood Smear Images. JAMA Netw. Open.

[14] Maturana C.R., de Oliveira A.D., Nadal S., Serrat F.Z., Sulleiro E., Ruiz E., Bilalli B., Veiga A., Espasa M., Abelló A. (2023). iMAGING: A novel automated system for malaria diagnosis by using artificial intelligence tools and a universal low-cost robotized microscope. Front. Microbiol..

[15] Maturana C.R., de Oliveira A.D., Zarzuela F., Ruiz E., Sulleiro E., Mediavilla A., Martínez-Vallejo P., Nadal S., Pumarola T., López-Codina D. (2024). Development of an automated artificial intelligence-based system for urogenital schistosomiasis diagnosis using digital image analysis techniques and a robotized microscope. PLoS Negl. Trop. Dis..

[16] Meulah B., Hoekstra P.T., Popoola S., Jujjavarapu S., Aderogba M., Fadare J.O., Omotayo J.A., Bell D., Hokke C.H., van Lieshout L. (2025). Evaluation of the AiDx Assist device for automated detection of Schistosoma eggs in stool and urine samples in Nigeria. Front. Parasitol..

[17] Lundin J., Suutala A., Holmström O., Henriksson S., Valkamo S., Kaingu H., Kinyua F., Muinde M., Lundin M., Diwan V. (2024). Diagnosis of soil-transmitted helminth infections with digital mobile microscopy and artificial intelligence in a resource-limited setting. PLoS Negl. Trop. Dis..

[18] Mathison B.A., Kohan J.L., Walker J.F., Smith R.B., Ardon O., Couturier M.R. (2020). Detection of Intestinal Protozoa in Trichrome-Stained Stool Specimens by Use of a Deep Convolutional Neural Network. J. Clin. Microbiol..

[19] Mathison B.A., Knight K., Potts J., Black B., Walker J.F., Markow F., Wood A., Bess D., Dixon K., Cahoon B. (2025). Detection of protozoan and helminth parasites in concentrated wet mounts of stool using a deep convolutional neural network. J. Clin. Microbiol..

[20] Bradbury R.S., Sapp S.G.H., Potters I., Mathison B.A., Frean J., Mewara A., Sheorey H., Tamarozzi F., Couturier M.R., Chiodini P. (2022). Where Have All the Diagnostic Morphological Parasitologists Gone?. J. Clin. Microbiol..

