# Supplementary material for: Validation of Digital Slide Scanning and a Convolutional Neural Network for the Detection of Intestinal Parasites in Human Stool Samples
Source: Diagnostics (Basel). 2025 Nov 24;15(23):2974. doi: 10.3390/diagnostics15232974 (PMC12691387; doi:10.3390/diagnostics15232974)
Supplement: Supplementary file 1 [file diagnostics-15-02974-s001.zip › diagnostics-3963526-supplementary.pdf]

Supplementary data

Table S1: Comparison of stool concentration devices. Results are displayed in following order: correct pre-classification (yes/no), correct classification (yes/no), quantification (+ ≤ 1 organism/high-power field (0.4 mm^2), ++ 1-9 organism/high-power field (0.4 mm^2), +++ ≥ 10 organism/high-power field (0.4 mm^2)). n.a.= not applicable.

sample	category/species	Mini Parasep SF	StorAX
1	*Ascaris lumbricoides*	yes/yes/+	yes/yes/++
2	*Enterobius vermicularis*	yes/yes/+	yes/yes/+
3	Hookworm / *Trichostrongylus*	yes/yes/+	yes/yes/++
4	*Schistosoma mansoni*	yes/yes/+	yes/yes/+
5	*Taenia* spp.	yes/yes/+	yes/yes/++
6	*Blastocystis* spp.	yes/yes/++	yes/yes/+++
7	*Blastocystis* spp.	yes/yes/+	yes/yes/++
8	*Endolimax nana*	yes/yes/+++	yes/yes/+++
9	*Endolimax nana*	yes/yes/+	yes/yes/++
10	*Entamoeba coli* (*Entamoeba* spp.)	yes/yes/+	yes/yes/+
11	*Entamoeba coli* (*Entamoeba* spp.)	yes/yes/++	yes/yes/+++
12	*Entamoeba histolytica/dispar* (*Entamoeba* spp.)	yes/yes/+	yes/yes/+
13	*Entamoeba histolytica/dispar* (*Entamoeba* spp.)	yes/yes/+	yes/yes/++
14	*Giardia duodenalis*	yes/yes/++	yes/yes/+++
15	*Giardia duodenalis*	yes/yes/++	yes/yes/++
16	*Blastocystis* spp.<br>*Iodamoeba buetschlii*	yes/yes/++<br>yes/yes+	yes/yes/++<br>yes/yes+
17	*Blastocystis* spp.<br>*Iodamoeba buetschlii*	yes/yes/++<br>yes/yes+	yes/yes/++<br>yes/yes++
18	*Entamoeba hartmanni* (MSP),<br>*Entamoeba histolytica/dispar* (*Entamoeba* spp.)<br>*Iodamoeba buetschlii*	yes/yes/+<br>yes/yes/+<br>yes/yes/+	yes/yes/++<br>yes/yes/+<br>yes/yes/++
19	*Blastocystis* spp.<br>*Entamoeba histolytica/dispar* (*Entamoeba* spp.)<br>*Iodamoeba buetschlii*	yes/yes/+<br>yes/yes/+<br>yes/yes/+	yes/yes/+<br>yes/yes/+<br>yes/yes/++
20	*Blastocystis* spp.<br>*Iodamoeba buetschlii*	yes/yes/++<br>yes/yes/+	yes/yes/+++<br>yes/yes/++
21-30	negative	no/no/n.a.	no/no/n.a.

Table S2: Intra-run precision with three scans within a day.

sample	scan 1	scan 2	scan 3
1	*A. lumbricoides*	*A. lumbricoides*	*A. lumbricoides*
2	*G. duodenalis*	*G. duodenalis*	*G. duodenalis*
3	negative	negative	negative

Table S3: Inter-run precision with a scan on five consecutive days.

sample	scan 1	scan 2	scan 3	scan 3	scan 4	scan 5
1	*A. lumbricoides*	*A. lumbricoides*	*A. lumbricoides*	*A. lumbricoides*	*A. lumbricoides*	*A. lumbricoides*
2	*G. duodenalis*	*G. duodenalis*	*G. duodenalis*	*G. duodenalis*	*G. duodenalis*	*G. duodenalis*
3	negative	negative	negative	negative	negative	negative
